# Antibiotic dispensing practices during COVID-19 and implications for antimicrobial resistance (AMR): parallel mystery client studies in Uganda and Tanzania

**DOI:** 10.1186/s13756-022-01199-4

**Published:** 2023-02-11

**Authors:** Emmanuel Olamijuwon, Eveline Konje, Catherine Kansiime, Mike Kesby, Katherine Keenan, Stella Neema, Benon Asiimwe, Stephen E. Mshana, Martha F. Mushi, Olga Loza, Benjamin Sunday, Alison Sandeman, Derek J. Sloan, Fernando Benitez-Paez, Joseph R. Mwanga, Wilber Sabiiti, Matthew T. G. Holden, CARE Consortium, Joel Bazira, Christine Muhumuza, Ivan Muhwezi, Kathryn Jean Fredricks

**Affiliations:** 1 School of Geography and Sustainable Development, University of St Andrews, St Andrews, KY16 9AL UK; 2 Department of Biostatistics and Epidemiology, School of Public Health, Catholic University of Health and Allied Sciences, P.O. Box 1464, Mwanza, Tanzania; 3 School of Public Health, College of Health Sciences, Makerere University, Kampala, Uganda; 4 Department of Sociology and Anthropology, Makerere University, Kampala, Uganda; 5 Department of Medical Microbiology, Makerere University College of Health Sciences, Kampala, Uganda; 6 Department of Microbiology, Mbarara University of Science and Technology, Mbarara, Uganda; 7 School of Medicine, University of St Andrews, St Andrews, KY16 9AL UK; 8 Department of Microbiology, Catholic University of Health and Allied Sciences, P.O. Box 1464, Mwanza, Tanzania

**Keywords:** COVID-19, Uganda, Tanzania, Drug sale, Pharmacy, Antimicrobial resistance, Mystery client

## Abstract

**Background:**

Over-the-counter antibiotic access is common in low-and-middle-income countries and this may accelerate antimicrobial resistance. Our study explores critical aspects of the drug seller–client interaction and antibiotic dispensing patterns for simulated COVID-19 symptoms during the pandemic in two study sites in Tanzania and Uganda, countries with different government responses to the pandemic.

**Methods:**

Research assistants posing as clients approached different types of drug sellers such as pharmacies (Pharms), drug shops (DSs), and accredited drug dispensing outlets (ADDOs) in Mwanza, Tanzania (*nPharms* =  415*, nADDOs* = 116) and Mbarara, Uganda (*nPharms* = 440, *nDSs * = 67), from June 10 to July 30, 2021. The mystery clients held no prescription and sought advice for simulated COVID-19 symptoms from the drug sellers. They documented the quality of their interaction with sellers and the type of drugs dispensed.

**Results:**

Adherence to COVID-19 preventive measures and vigilance to COVID-19 symptoms was low in both sites but significantly higher in Uganda than in Tanzania. A higher percentage of drug sellers in Mbarara (Pharms = 36%, DSs = 35%, *P*-value = 0.947) compared to Mwanza (Pharms = 9%, ADDOs = 4%, *P*-value = 0.112) identified the client’s symptoms as possibly COVID-19. More than three-quarters of drug sellers that sold prescription-only medicines in both Mbarara (Pharms = 86%, DSs = 89%) and Mwanza (Pharms = 93%, ADDOs = 97%) did not ask the MCs for a prescription. A relatively high percentage of drug sellers that sold prescription-only medicines in Mwanza (Pharms = 51%, ADDOs = 67%) compared to Mbarara (Pharms = 31%, DSs = 42%) sold a partial course without any hesitation. Of those who sold antibiotics, a higher proportion of drug sellers in Mbarara (Pharms = 73%, DSs = 78%, *P*-value = 0.580) compared to Mwanza (Pharms = 40% ADDOs = 46%, *P*-value = 0.537) sold antibiotics relevant for treating secondary bacterial infections in COVID-19 patients.

**Conclusion:**

Our study highlights low vigilance towards COVID-19 symptoms, widespread propensity to dispense prescription-only antibiotics without a prescription, and to dispense partial doses of antibiotics. This implies that drug dispensing related to COVID-19 may further drive AMR. Our study also highlights the need for more efforts to improve antibiotic stewardship among drug sellers in response to COVID-19 and to prepare them for future health emergencies.

**Supplementary Information:**

The online version contains supplementary material available at 10.1186/s13756-022-01199-4.

## Introduction

Notwithstanding the lower rate of testing and potential under-reporting of COVID-19 cases, the direct impacts of COVID-19, in terms of morbidity and mortality, have been less severe in Africa compared with other world regions [1]. This may be attributable to a number of factors, such as younger age structures, African governments’ responses, and health system preparedness for infectious diseases [2–6]. However, COVID-19 will likely have many serious indirect impacts on health and society in Africa. An area of particular concern is how COVID-19 response and preventative measures, and their unintended consequences, will affect the pre-existing and ongoing ‘silent pandemic’ of antimicrobial resistance (AMR). AMR, like COVID-19, is a global health threat that requires urgent attention [7]. Evidence suggests that the burden of AMR in low and middle-income countries (LMICs) is high compared to high-income countries [8]. There is a pressing need to understand the social factors that facilitate the biological development of AMR [9]. Among multiple social drivers, the motivations and behaviours of drug sellers and pharmacists play a critical role in AMR because they impact how people access antibiotics in LMICs, and because inappropriate dispensing practices may fuel drug resistance [10–12]. This paper investigates drug sellers’ practices during the COVID-19 pandemic in sites in two countries (Mbarara, Uganda and Mwanza, Tanzania) with varying societal responses to the pandemic.

How the COVID-19 and AMR pandemics are interacting is unclear and likely to be context-dependent. A significant potential risk in LMICs is that COVID-19 prevention policies may drive an increase in antibiotic self-medication through over-the-counter purchases and a breakdown in antibiotic stewardship [13]. Patients may avoid healthcare facilities when seeking treatment for other ailments, for fear of catching COVID-19 in these settings, or may be reluctant to present with COVID-19-like symptoms [14–16]. Interruptions in public healthcare provision for illnesses including HIV, malaria, and tuberculosis may have increased footfall at pharmacies and drug shops, which have remained open during lockdowns as essential services [17]. Finally, the COVID-19 ‘infodemic’ [18], particularly the aspect which promotes misleading information about antibiotic use to prophylactically ‘treat’ COVID-19 [19, 20], may have also increased demand for the over-the-counter sale of antibiotics [21, 22]. The health impact of this ‘infodemic’ could even be worse given that they are circulated on various media platforms and promoted by a several authorities, from traditional and religious leaders to politicians on the world stage. Alternatively, an increased focus on public health, risk avoidance, and public health safety regulations may have improved antibiotic stewardship and dispensing practices.

In both Tanzania and Uganda, drug sellers operate under well-established regulatory frameworks. In Tanzania, they are comprised of two main types: type 1 pharmacies, which are supervised by a registered pharmacist and are permitted to sell a wide range of prescription-only medications, including antibiotics; and type 2 (known officially as accredited drugs dispensing outlets—ADDOs—and colloquially as ‘maduka ya dawa muhimu’, literally, ‘essential medicines shops’), which a suitably qualified^1^ person can run after completing the 5-week ADDO training course, but which can dispense a more limited range of drugs. ADDOs were established in 2003 as part of a national policy to address shortages in fully-qualified pharmacists and increase access to pharmaceutical drugs, including antimicrobials [23]. In 2019 there were 1504 type 1 registered pharmacies and 14,045 ADDOs in Tanzania [24]. Chalker et al. [25] found that respondents favoured the latter over public health facilities as their preferred means to access antimicrobials [25]. Uganda has two main types of drug sellers: Type 1 pharmacies, which are supervised by a qualified pharmacist and permitted to sell a wide range of prescription-only medications; and drug shops, which are managed by individuals with at least 2 years of health training or a health-related qualification and which can sell a limited range of medicines [26]. The small retail drug shops generally have minimal health infrastructure and often sell pharmaceutical drugs alongside other general services [26]. They are often the main source of pharmaceutical drugs in rural areas [27, 28]. In both countries, private drug shops and ADDOs are regulated and inspected before approval. This should ideally continue after approval, but is often limited by financial constraints [29, 30].

Despite clear regulations stating that a prescription is required before antibiotics are dispensed, over-the-counter sales are common in many LMICs [26, 31–33]. Mbonye et al. [31] showed that the majority of drug shops in Uganda sold antibiotics, especially amoxicillin and trimethoprim-sulfamethoxazole, even though they were not authorised to do so [34]. Drug sellers in Uganda were aware of national regulations and rarely displayed prohibited drugs on the shelf for fear of inspectors and penalties, but would provide the client with the same on demand [26]. Mystery client studies conducted as part of our consortium’s earlier work on AMR recorded widespread non-compliance with regulations prohibiting antibiotic sales without a prescription in Tanzania [32, 35]. The study found that most providers across three study regions dispensed amoxicillin on-demand without question, to clients with no prescription [32].

There is little evidence, however, of how antibiotic prescribing and dispensing have been affected by the COVID-19 pandemic, especially from Sub-Saharan Africa, including Tanzania and Uganda. This study builds on an earlier, pre-COVID-19, project on the drivers of AMR in East Africa [36] to explore pandemic-era drug seller behaviours and practices in Tanzania and Uganda. The study was conducted in mid-2021, when healthcare facilities were struggling to cope with COVID-19 alongside the usual burden of disease, mitigation measures were still evolving, and both governments and citizens were attempting to adjust to the unprecedented conditions. This study investigates the following questions: To what extent do drug sellers in the different settings follow internationally recommended best practices for COVID-19 infection control related to hygiene, mask use and social distancing? How vigilant are drug sellers to COVID-19 like symptoms and what is the nature and quality of advice they give to clients presenting with such symptoms? Do drug sellers recommend antibiotics to clients reporting COVID-19 like symptoms?

Comparing drug dispensing practices in Uganda and Tanzania is important because although their antibiotic provision landscapes were similar pre-COVID-19, their early COVID-19 policy response diverged considerably in ways that may have shaped individual risk assessment, compliance with safety guidelines, and susceptibility to AMR [37]. During the pandemic, Uganda implemented stringent household lockdowns and social distancing regulations, including restrictions on public travel and closure of non-essential shops and markets. Tanzania’s approach was less consistent: fluctuating from strict public health measures being in place (March–April 2020), through a period of so-called ‘COVID denial’ (April 2020—early 2021) in which public health measures and testing capacity were rolled back [38, 39]. Before 2021, Tanzania was one of the few countries that imposed few physical distancing measures [37, 40]. Although residents were advised against physical contacts, such as shaking hands, there have been no major lockdowns or closures of non-essential activities in order to protect the country’s economy, and no restrictions on public gatherings for religious activities [37, 40]. Misinformation has also been prevalent in Tanzania. For example, spiritual remedies through prayers were promoted as strategies for combatting the pandemic [37, 39]. With new leadership (March 2021—present) the position is of stricter measures to curb the spread of the virus, such as the enforcement of mass masking mandates and the establishment of new testing centres.

## Methods

### Study design

This cross-sectional community-based quantitative survey is part of the study CARE: COVID-19 and Antimicrobial Resistance in East Africa, which built on an interdisciplinary study of drivers of AMR in East Africa (Holistic Approach to Unravel Antibacterial Resistance in East Africa or HATUA), details published elsewhere [36]. We used a mystery client design, also referred to as a simulated client study, to assess drug-dispensing practices. Mystery client studies are commonly used to observe drug provider behaviour while minimising observation bias [25, 32]. In our study, 32 male and female field workers who were 4th year pharmacy students (in Tanzania) or bachelor’s degree holders (in Uganda), unknown to the sellers, posing as clients (hereafter known as mystery clients, or MCs) visited the pharmacies, ADDOs and drug shops.

MCs presented with COVID-19 like symptoms, reporting fever, cough, sore throat, loss of sense of taste and smell. They were provided with suggestions on how to respond during the encounter with drug sellers (full details in the Additional file 1: S1). If asked further about symptoms, the MC replied that this was the first time they had experienced them (developed in the last 48 h), that they had not visited a doctor, nor did they have a prescription, nor were they taking any other drugs. If offered any kind of test (for COVID-19 or others), they declined. If asked about their knowledge of COVID-19, they reported some knowledge but general confusion about symptoms. If the seller did not recommend a pharmaceutical drug, the MC asked why they could not have amoxicillin (the drug they ‘usually took for such symptoms’) or even ‘something stronger’ since they were ‘experiencing a lot of pain’ (a statement designed to prompt sellers to challenge clients about the use and utility of antibiotics for the treatment of respiratory symptoms/pain and about the need for a prescription). If offered any pharmaceutical drug, the MC made one attempt to purchase ‘only a couple of days’ worth to see if it worked’. This response was designed to elicit sellers’ advice that a full course of antibiotics should be purchased and consumed. The scenarios were developed following previous MCSs, co-designed with local academics and clinicians. All scenarios were piloted for feasibility and local sensitivity.

Immediately after each encounter, MCs retired to a less public space to record their interactions with the drug sellers on mobile devices installed with Epicollect5 [41], before moving to the next seller (see Additional file 2: S2 for a copy of the questionnaire). This approach allowed the MCs to capture data on mobile devices while the memory was still fresh without jeopardising the quality of information from the drug sellers. To reduce variability between the response and scenarios, the MCs participated in a series of training sessions and fieldwork pilots in which the reliability of the responses captured were extensively assessed. The MCs were also instructed to follow the scenarios strictly without improvising. In addition, the MCs have prior knowledge of conducting simulated client studies in these settings [32].

Furthermore, to minimise the potential spread of the virus during fieldwork, it was mandatory for all MCs to wear a face mask during interaction with drug sellers, have a personal sanitiser and use it before entering the premises and after each interview, keep a safe distance and avoid physical contact (handshaking, hugging etc.). They were, however, not tested for COVID-19 before and during the data collection period. This study has been reported in accordance with the Checklist for Reporting Research using Simulated Patient [42] methodology (see Additional file 3: S3).

### Data collection and sample

**Fig. 1:**
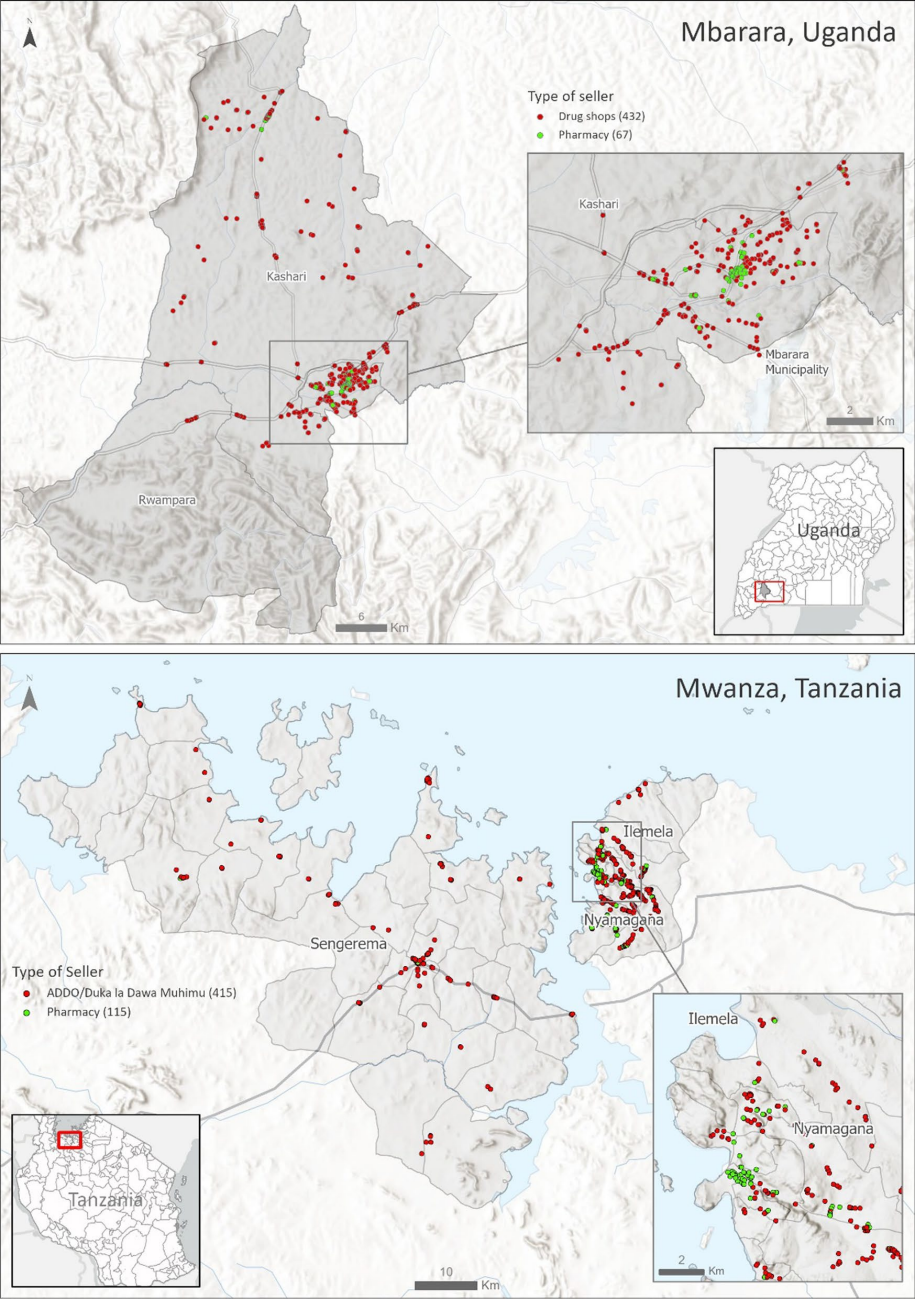
Geographic distribution of drug sellers sampled in the survey

The sample frame built upon earlier work undertaken as part of the 3-country HATUA study, in which Mwanza district in Tanzania and Mbarara district in Uganda were two of nine sites. Between April and July 2019, we comprehensively mapped drug outlets within Mwanza and Mbarara districts to create a sample frame for mystery client studies [32, 35]. Trained field assistants systematically traversed roads and pedestrian areas, using Global Positioning System (GPS) enabled tablets and phones and the software Epicollect5 [41] to record location and basic descriptive details about formal and informal outlets that sold medicine for humans [36]**.** In 2019, 427 drug shops and 271 pharmacies were recorded in Mbarara in Uganda, while 508 ADDOs and 113 pharmacies were recorded in Mwanza, Tanzania [32]. We aimed to resurvey all of the original sample frame, with a minimum target of 500 of the original drug shops/ADDOs/pharmacies in each sites. We resurveyed 507 pharmaceutical drug sellers in Mbarara (440 drug shops and 67 pharmacies) and 531 pharmaceutical drug sellers in Mwanza (415 ADDOs and 67 pharmacies) between 10 June and 30 July 2021. Outlets were visited once by one MC. As well as those in the original sample frame, we included any new outlets that were operating and open during data collection period. Figure 1 presents the spatial distribution of drug sellers included in this study. Before conducting the study, the CARE team distributed leaflets to all ADDOs, drug shops, and pharmacies in both countries explaining the research aims and objectives and inform sellers of the intended survey within 90 days of flyer distribution (see Additional file 4: S4).

### Study variables

During data collection, we recorded general information about the drug sellers, including the seller type, type of ownership, affiliation, and size of shop. In this study, the shop size was considered small or large based on room size and presence of a counter with capacity for receiving 3 or more clients while keeping social distancing. All types of outlets are required to be clean and to have their licence and qualifications displayed [29, 43]. The MCs observed the environment of the drug outlets and recorded whether a licence was visible in the shop, handwashing facilities, hand sanitiser and COVID-19 health information were visible around the shop, and whether intentional or accidental social distancing was observed.

MCs also recorded data on a range of issues. First, regarding vigilance: whether sellers suggested explicitly that the symptoms described might be COVID-19 and/or demonstrated any non-verbal signs (shock or alarm) that they were alert to this possibility. Second, regarding drug sellers’ response: whether sellers asked if the symptoms were new or experienced previously, if a doctor had been consulted, and if the MC had a prescription or was taking other medications. Third, regarding advice provided: whether specific COVID-19 related advice was given during the encounter, including advice to get tested for COVID-19, self-isolate, maintain social distancing and/or wear a face mask as a safety precaution to protect others. Fourth, regarding dispensing practice: whether an antibiotic was sold, whether the MC or the seller had first suggested this, and whether the seller responded positively to the MC’s request for only a couple of days’ worth of medicine. If the seller agreed to sell ‘a few days’ worth’, we considered this as ‘sold a partial course’ (see Additional file 1: S1 for a detailed description of the average number of tablets sold as ‘a few days’ worth/ partial course’). Situations where sellers granted the client their wish for ‘a few days’ worth but advised them to buy and finish a full course were classified as ‘sold partial course with a recommendation of finishing full course’. The drug names and the number of pills dispensed were recorded in all cases. Finally, MCs recorded whether the drug seller gave clear instructions that a full minimum course should be taken even if they did not sell one on this occasion.

Data on the drugs sold were categorised in three ways: first, as either ‘prescription-only’ or ‘over-the-counter’ (if national regulations meant they could be sold without a prescription). Second, using the World Health Organisation (WHO) AWaRe classification of ‘access’, ‘watch’, and ‘reserve’ [44]. Third, we identified which drugs had been named in national guidelines as suitable for treating patients with confirmed, severe COVID-19 to control secondary bacterial co-infections.

### Patient and public involvement

We worked closely with government, research agencies and community stakeholders during the design, and data collection phase of the study through inception and dissemination workshops that involved local government authorities, district and regional health management teams, and the pharmacy council. Preliminary findings have also been shared in a joint stakeholders’ engagement meeting, a process which, in turn, fed into the analysis and interpretation of the data.

### Statistical analysis

All responses from the fieldwork were retrieved from Epicollect5 and imported into R Studio for further cleaning, analysis, and visualisation. Frequency and percentage distributions were used to describe characteristics of drug sellers, the quality of interaction between MCs and drug sellers, measures of compliance with COVID-19 safety precautions and other patterns that emerged from the data. All analyses were stratified by drug seller type to delineate differences in drug dispensing practices between drug shops/ADDOs and pharmacies. We used the chi-square test or Fisher exact test (in the case of small cell counts) to assess significant differences in drug dispensing practices between drug shops/ADDOs and pharmacies in both countries.

## Results

### Characteristics of the drug outlets and COVID-19 preventative measures

Table 1 presents a summary of the characteristics of the drug outlets surveyed. In both study sites, most pharmacy outlets sampled were considered ‘large’ or ‘wholesale’ pharmacies, while most drug shops or ADDOs were characterised as ‘small’ shops. In both study sites, pharmacies were more likely to display registration licences or certificates than drug shops or ADDOs. More than half of drug shops (51%) and pharmacies (70%) surveyed in Mbarara, Uganda, had hand sanitiser available, compared with less than one-tenth of ADDOs (5%) and pharmacies (7%) surveyed in Mwanza, Tanzania. More than half of all drug seller types in both settings, however, have a handwashing facility. COVID-19 information was visible in 48% of Ugandan outlets, compared with only 7% of Tanzanian ones.

**Table 1:** Descriptive Characteristics of the Drug Sellers in Tanzania and Uganda

Characteristics	Mbarara, Uganda N (column %)			Mwanza, Tanzania N (column %)		
	Pharmacy	Drug shops	All sellers	Pharmacy	ADDO	All sellers
*Size of the facility*						
Large/wholesale pharmacy	57 (85.1)	13 (3.0)	70 (13.8)	84 (72.4)	6 (1.4)	90 (16.9)
Small shop	10 (14.9)	427 (97.0)	437 (86.2)	32 (27.6)	409 (98.6)	441 (83.1)
*License and certification*						
License/certificate not visible	13 (19.4)	133 (30.2)	146 (28.8)	9 (7.8)	164 (39.5)	173 (32.6)
License/certificate visible	54 (80.6)	307 (69.8)	361 (71.2)	107 (92.2)	251 (60.5)	358 (67.4)
*Presence of hand sanitizer*						
Hand sanitizer not present	20 (29.9)	218 (49.5)	238 (46.9)	108 (93.1)	396 (95.4)	504 (94.9)
Hand sanitizer present	47 (70.1)	222 (50.5)	269 (53.1)	8 (6.9)	19 (4.6)	27 (5.1)
*Presence of hand washing facility*						
Handwashing facility not present	13 (19.4)	34 (7.7)	47 (9.3)	57 (49.1)	194 (46.7)	251 (47.3)
Handwashing facility present	54 (80.6)	406 (92.3)	460 (90.7)	59 (50.9)	221 (53.3)	280 (52.7)
*Visibility of COVID-19 health information*						
COVID-19 health information not visible	34 (50.7)	231 (52.5)	265 (52.3)	101 (87.1)	395 (95.2)	496 (93.4)
COVID-19 Health information visible	33 (49.3)	209 (47.5)	242 (47.7)	15 (12.9)	20 (4.8)	35 (6.6)
Total	67	440	507	116	415	531

### Vigilance to COVID-19 symptoms and implementation of international best practices on transmission avoidance

**Fig. 2:**
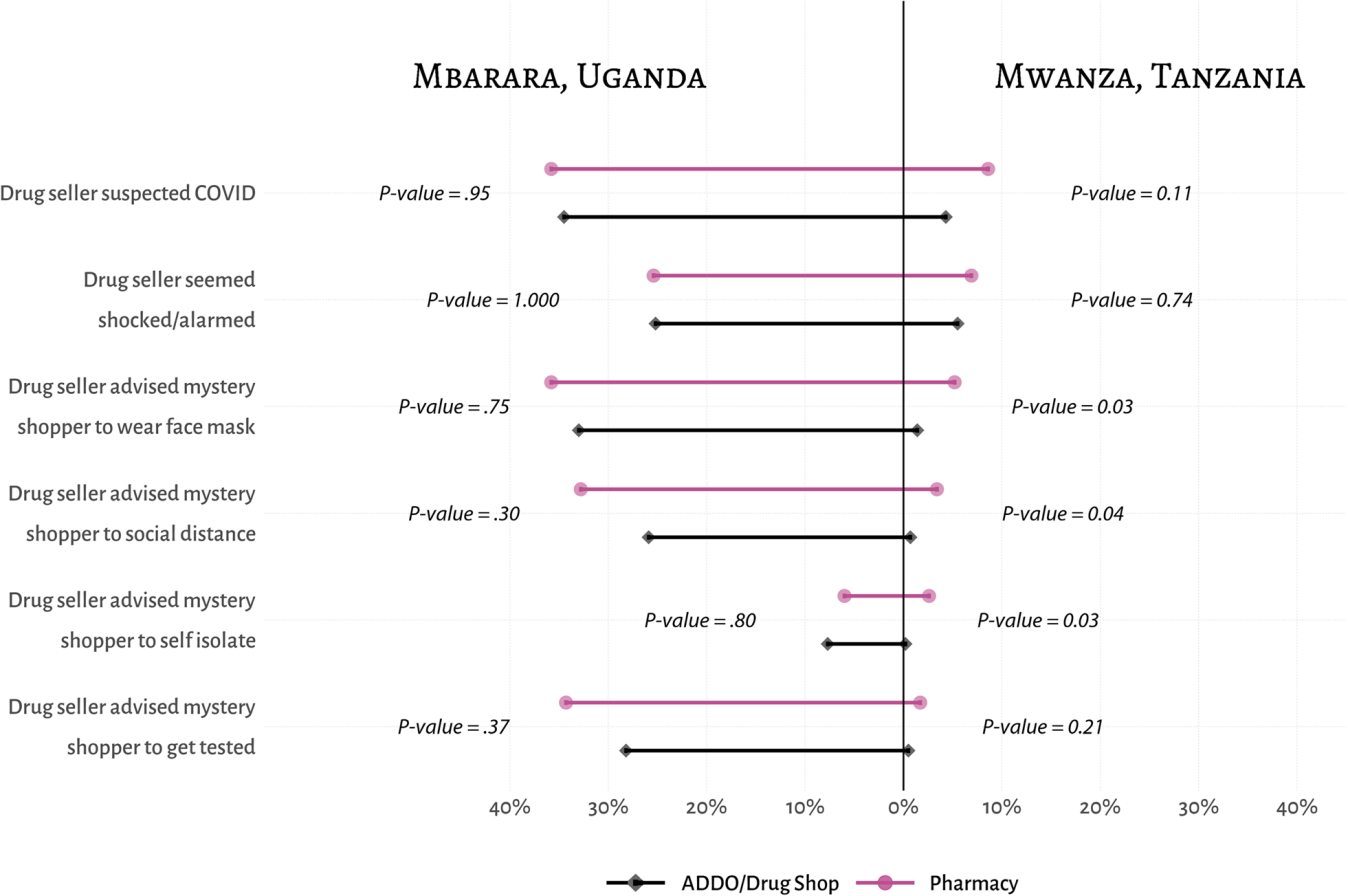
Explicit and implicit vigilance to COVID-19 and recommendations for managing COVID-19-like symptoms between drug seller types in Mbarara, Uganda and Mwanza, Tanzania

The summary of seller’s vigilance to COVID-19 symptoms and adherence to international guidance for preventing COVID-19 is presented in Fig. 2. On all measures, drug sellers in Mbarara, Uganda were more alert to the potential threat of COVID-19 and were more likely to advise COVID-19 prevention measures than their counterparts in Mwanza, Tanzania. Only about 9% of pharmacies and 4% of ADDOs in Mwanza, Tanzania explicitly suggested that MCs might have COVID-19, compared to more than a third of the pharmacies and drug shops in Mbarara, Uganda (36% and 35% respectively). More sellers in Mbarara, Uganda were also ‘surprised, shocked or alarmed’ at the mention of COVID-19 symptoms than those in Mwanza, Tanzania.

While there were small differences in the advice given by different outlet types within the sites, cross-national differences were more striking. A significantly higher percentage of drug sellers in Mbarara, Uganda compared to Mwanza, Tanzania gave MCs some recommendations for limiting the spread of COVID-19, such as testing, wearing a face mask, social distancing, and self-isolation. In Mwanza, Tanzania, drug sellers rarely implemented any common COVID-19 prevention practices, such as advising MCs to get tested, self-isolate, socially distance or wear a face mask. Less than 1% of drug sellers in ADDOs advised MCs to get tested, self-isolate, observe social distancing, or wear a face mask, compared to about 5% (or less) of pharmacies in the same study site. In Mbarara, Uganda, nearly a third of all drug sellers gave precautionary advice for limiting the spread of COVID-19. Among drug shops, 28% advised MCs to get tested, 8% to self-isolate, 26% to observe social distancing and 33% to wear a face mask.

### Dispensing practices and quality of drug seller-client interaction

**Fig. 3:**
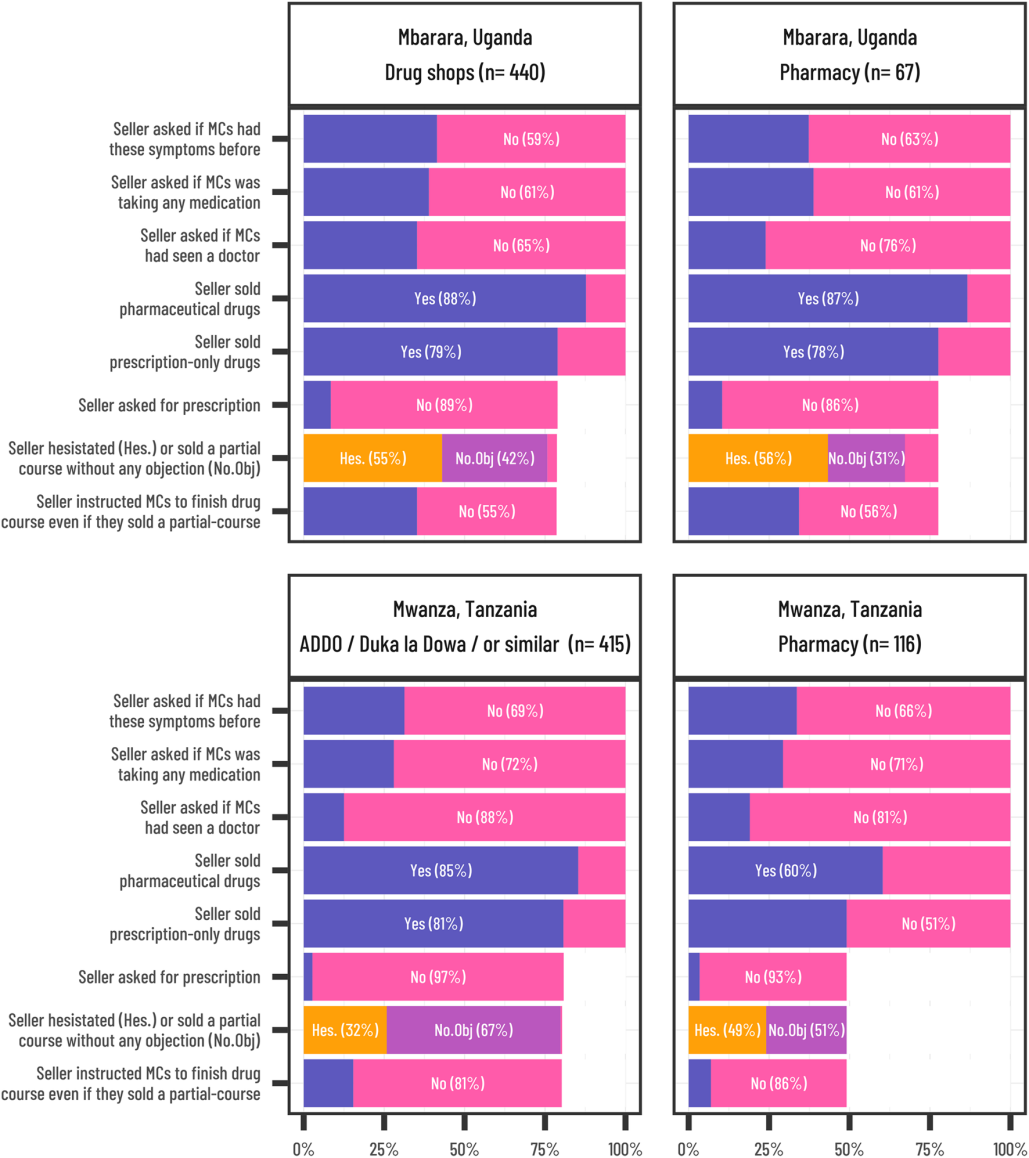
Patterns and quality of drug seller-client interaction by the quality of assessment, drug dispensing practices and advice. *Note*: No.Obj. = drug seller sold a partial course without any objection, Hes. = drug seller hesitated in selling half-course but still sold it

Figure 3 describes the attempts made to establish ‘patient history’ and the dispensing practices observed when sellers were presented with the study scenario. In Mbarara, Uganda, 41% of drug sellers asked MCs if they had experienced the symptoms before, compared to 32% in Mwanza, Tanzania. A minority of sellers asked if MCs had seen a doctor (12% of ADDOs and 19% of pharmacies in Mwanza, Tanzania and 25% of drug shops and 24% of pharmacies in Mbarara, Uganda). Critically, only about 3% of ADDOs and 7% of pharmacies in Mwanza, Tanzania asked the MC if they had a prescription compared to about 11% of drug shops and 14% of pharmacies in Mbarara, Uganda. In most cases, pharmaceutical drugs were sold; this was less common in pharmacies (60%) compared to ADDOs (85%) in Mwanza, Tanzania, but no such difference was observed between drug shops and pharmacies in Mbarara, Uganda (88% vs 87%). The majority of pharmaceutical drugs sold were prescription-only drugs.

Across both study sites, nearly 90% of drug sellers who sold prescription-only medicines agreed to the MCs request for ‘only a few days’ worth’ (recorded as ‘sold a partial course’). The situation was less common in Uganda, where 42% of drug shops and 31% of pharmacies that sold prescription-only medicines sold a ‘partial course’ without further questions. In Tanzania, however, 67% of ADDOs and 51% of pharmacies that sold prescription-only medicines sold a ‘partial course’ without challenging the MC’s request. In Uganda, 55% of drug shops and 56% of pharmacies that sold prescription-only medicines hesitated before dispensing a partial course, whereas in Tanzania only 32% of ADDOs and 49% of pharmacies did so. Drug sellers in Mbarara, Uganda were more likely to advise completing the course of prescription-only drugs sold (45% in both seller types) than those in Mwanza, Tanzania (19% of ADDOs and 14% of pharmacies). Analysis of the mean number of tablets sold (see Additional file 1: Table S1) shows that all sellers, regardless of the country and type of outlet, were likely to sell less than optimal numbers of tablets, and that mean numbers of tablets sold were lower in Mwanza, Tanzania than in Mbarara, Uganda.

### Characteristics of the drugs sold

**Fig. 4:**
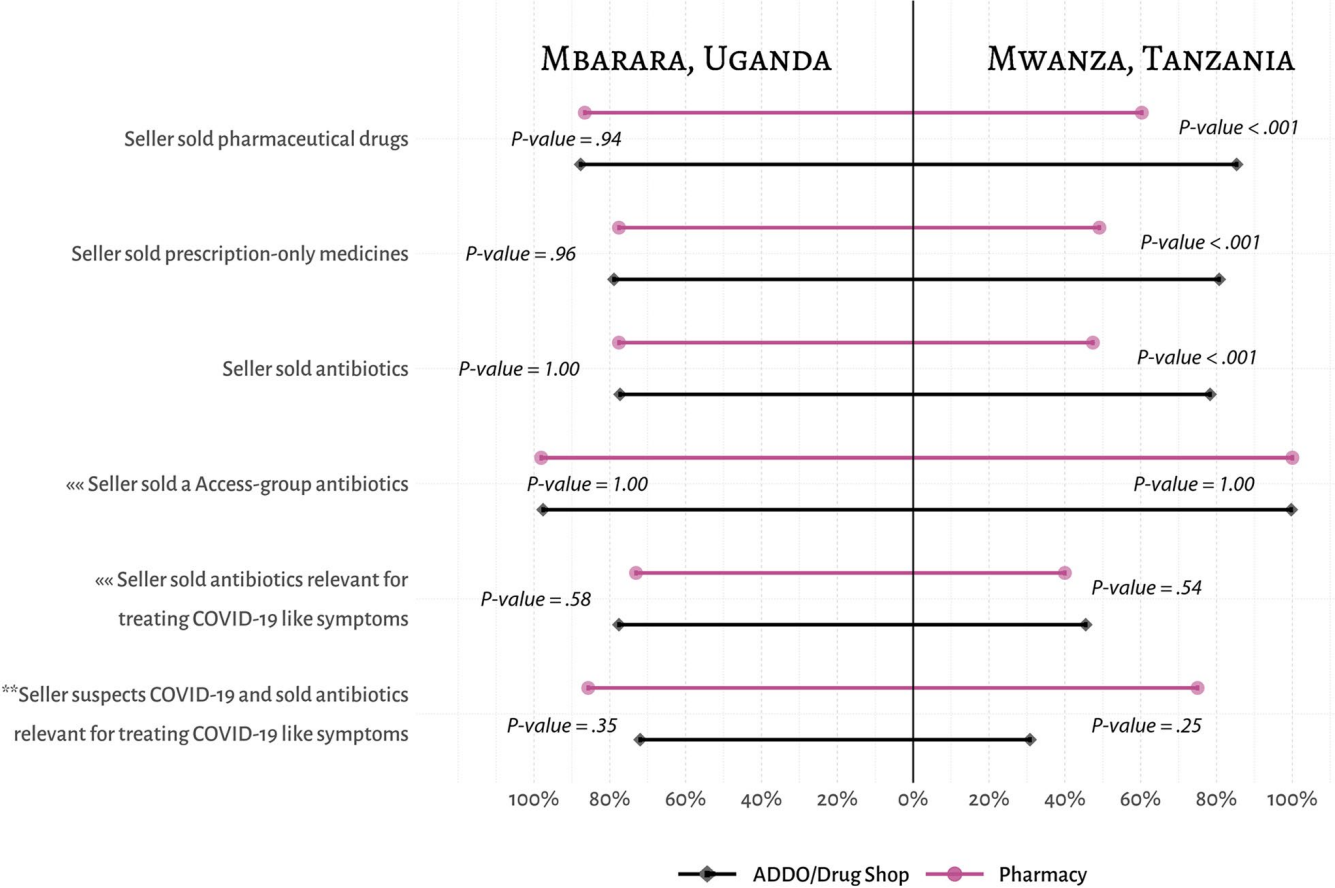
Comparison of drug-dispensing practices between pharmacies and drug shops/ADDOs in Tanzania and Uganda. Note: ««—implies that the percentage distribution is based on the total number of drug sellers who sold antibiotics; **—implies that the percentage distribution is based on the total number of drug sellers who suspected COVID-19 and sold antibiotics

The majority of prescription-only drugs were antibiotics (Fig. 4); a full list of the drugs dispensed by drug sellers and their WHO AWaRE classification are available in Additional file 1: S1. Of those sellers who sold antibiotics, nearly all sold drugs that were on the AWaRE ‘Access’ list, which are of the lowest concern in preventing the spread of AMR. Only one ADDO among all surveyed drug sellers in Mwanza, Tanzania sold an antibiotic on the WHO AWaRE ‘Watch’ list, which has a higher potential to lead to resistance, compared to about 2% of pharmacies and drug shops in Mbarara, Uganda. Nearly 80% of drug sellers in Mbarara, Uganda sold antibiotics that were compatible with national guidelines for treating secondary bacterial infections in COVID-19 patients, compared to less than half of the pharmacies (40%) and ADDOs (46%) in Mwanza, Tanzania. This distribution was, however, higher among drug sellers that suspected COVID-19. About 75% of pharmacies that sold antibiotics and suspected COVID-19 in Tanzania sold antibiotics listed as relevant for treating secondary bacterial infections in hospitalised patients with severe COVID-19.

## Discussion

Our study sought to investigate drug dispensing practices in Mbarara, Uganda and Mwanza, Tanzania, during the COVID-19 pandemic and to consider the implications of these practices for AMR. Mystery clients presented with COVID-19-like symptoms in two LMIC settings with different policy responses to the COVID-19 pandemic. Our analysis uncovers four main findings. First, drug sellers’ COVID-19 vigilance and prevention advice were more common in Uganda, which has experienced strict lockdowns and a more consistent policy response than in Tanzania. Nevertheless, given that MCs presented with potential COVID-19 symptoms during a pandemic, it is surprising that vigilance among sellers was low in both settings. Second, we observed poor compliance with antibiotic dispensing regulations in both sites and in all types of outlets. The majority of pharmacies and drug shops/ADDOs dispensed prescription-only antibiotics without a prescription and sold a partial course without without any recommendation to complete the drug course. This is similar to practices observed at the same sites in our earlier mystery client studies conducted pre-COVID-19 [32], and in other settings [26]. As research has shown, inappropriate consumption of antibiotics or consumption of partial courses has potentially wide-ranging implications for AMR [10–12]. Third, whether sellers interpreted the symptoms described as COVID-19 or a more common respiratory ailment, most dispensed an antibiotic for symptoms that could have been viral infection, which also has implications for inappropriate antibiotic consumption and AMR. Fourth, whilst most sellers in Mbarara, Uganda, and a large minority of those in Mwanza, Tanzania sold an antibiotic that was nationally approved for the treatment of secondary bacterial infections among patients suffering severe COVID-19 symptoms, MCs were undiagnosed and presented as suffering only mild symptoms. This suggests that even if the propensity to dispense antibiotics without prescription has not changed markedly, the pandemic may still increase inappropriate use of antibiotics, which may exacerbate AMR and therefore reduce the efficacy of drugs used for the treatment of bacterial infection, including secondary infection in COVID-19 and other upper respiratory tract infections.

It is clear from this study and others [26, 32, 33], that drug sellers in Uganda and Tanzania, like those in many other LMIC settings [10, 45–47], do not always adhere to regulations on antibiotic dispensing, even though many conformed to other aspects of licensing and regulation. Pharmacies, where sellers might be expected to have a higher degree of training and awareness of regulation, were only marginally better at following dispensing guidelines than drug shops or ADDOs. However, individuals holding the license may not always be the ones to operate the pharmacies or drug shops, as highlighted in several studies [48, 49]. This suggests that, in order to be effective, interventions should be aimed at all seller types, rather than targeting particular types of drug sellers.

Many existing studies suggest that the lack of legal consequences for inappropriate prescription and dispensing of antibiotics is a key factor for the dispensing practices we observed in this study [45, 50–52], which leads researchers to emphasise the need for the enforcement of regulations, stricter surveillance of drug sellers’ practices and more severe sanctions for poor practice. However, there is limited evidence that enforcement of regulations alone can successfully improve antibiotic stewardship outside of a suite of multifaceted measures aimed at all stakeholders, including sellers, upstream actors like pharmaceutical suppliers, and clients themselves [53]. Consistent with Goel, et al.’s conceptual framework [54], a plethora of factors drive drug seller behaviours in these settings. Drug sellers’ motivations to dispense include pressure from both patients and suppliers [8, 26, 48, 55], which emphasises the relationality of the encounter and the supply–demand dynamics at play. Pharmaceutical companies and their representatives are also likely to pressure, encourage or incentivise drug sellers to prescribe or dispense specific brands of antibiotics [52]. At the same time, drug sellers are also likely to have commercial interests in complying with their customers’ requests, including both the need to profit [56] and building goodwill to ensure repeat visits in the future [48]. Thus, further work should address the interrelated contextual determinants of drug seller practices in LMICs to design more effective interventions. One example would be upskilling and knowledge training among drug sellers. For example, Kitutu et al. [57] report an intervention in Uganda, which successfully motivated adherence to recognised guidelines by engaging drug sellers as partners to enhance effective treatment for uncomplicated malaria.

Lastly, our finding on the differential compliance with internationally recognised preventative measures for curtailing the spread of COVID-19 (such as handwashing and wearing a mask) reveals how national policy responses shaped individuals’ behaviours and perceptions of risk. Other studies in Tanzania have observed limited social distancing and preventative measures [37]. This contrasts with Uganda, where more stringent policies and public health messaging were adopted, including school closures, household lockdowns and hygiene measures. The Tanzanian government emphasised the protection of citizens’ livelihoods and therefore did not aim to fundamentally change their behaviour. This national response may have shaped population-level awareness of the virus.

This study is the first to investigate drug seller practices during the COVID-19 pandemic in Uganda and Tanzania using a mystery client approach. Despite its contributions, it is not without its limitations. First, although we used a comprehensive sample frame of sellers, ground-tested and used in other studies [32], we were not able to reach all of the original sample frame, and this was particularly challenging for pharmacies (many of which are connected to formal healthcare facilities/clinics) in Uganda under a strict household lockdown, which could have led to sampling bias. Relatedly, although the study sample comprised one region in each country, and hence might not be generalisable, these regions did comprise both urban and rural areas, and our previous study suggests that patterns of response in the two sites are mirrored in the other country sites [32]. Second, the study used a ‘mystery client’ approach which relied on MCs’ subjective assessment of their interactions with drug sellers. For example, MCs reported whether drug sellers seemed shocked or alarmed at the mention of COVID-19 symptoms. They may not have been able to adequately capture the sellers’ reaction because some drug sellers, particularly in Tanzania, may have avoided mentioning COVID-19, even if they suspected it, to avoid potential repercussions for themselves and their business, particularly in settings characterised by high COVID-19 denial. Consequently, there may be a possibility that this study undercounted the sellers who suspected COVID-19. In addition, the scenario of presenting with respiratory symptoms may have been challenging to simulate realistically, leading sellers to suspect a milder ailment. Finally, MCs in Tanzania did not include the ‘loss of taste/smell’ in their symptom presentation to the drug sellers because, at the time, this was not locally recognised as a COVID-19 symptom. This may have affected the sellers’ response to MCs, including the likelihood of suspecting COVID-19, the advice given, and the drugs dispensed. Nonetheless, MCs presented with upper respiratory tract symptoms in the midst of a global viral respiratory pandemic; therefore, it would be reasonable to expect drug sellers to recognise the possibility of COVID-19 infection, which therefore upholds the validity of our results.

## Conclusion and implications

Drug sellers play a vital role in providing the public with access to antibiotics in LMIC settings. This study confirms that the patterns of antibiotic dispensing by drug sellers observed previously, which potentially place both patients and medicines at risk, have not altered during the COVID-19 pandemic. When presented with symptoms commensurate with COVID-19/upper respiratory tract infection, with no indication of bacterial origin, drug sellers commonly sold antibiotics without a prescription. This suggests that, as COVID-19 spreads in communities and more people present with such symptoms, (inappropriate) antibiotic dispensing may become more frequent, which may further drive AMR, including in drugs vital for treating secondary infections in COVID-19 patients. Moreover, despite MCs presenting with COVID-19-like symptoms, the sellers suspected COVID-19 in very few cases and did not recommend infection prevention measures, which may have implications for COVID-19 transmission.

Furthermore, many sellers dispensed prescription-only antibiotics approved for treating bacterial co-infection more likely to manifest in patients experiencing *severe* COVID-19. Such prophylactic use is a misapplication in both clinical and regulatory terms and opens new avenues for the development of AMR. Overall, our findings suggest that existing systems designed to regulate antibiotic use, prevent AMR, and halt the spread of COVID-19 are not robust. To address this, recognising drug sellers’ unique and indispensable role as first points of call in these communities may be equally as productive as restriction and sanction. Drug sellers in LMICs are uniquely positioned to act as an important first-line protection mechanism against both COVID-19 spread, and inappropriate antibiotic dispensing. Solutions may come in thinking about drug sellers’ role in the healthcare system inclusively and using principles of co-production to develop policy and messaging around AB stewardship.

## Supplementary Information

**Additional file 1**. Copy of scripts given to MCs and supplementary analysis.

**Additional file 2**. Copy of data collection instrument.

**Additional file 3**. Checklist for reporting research using simulated patient.

**Additional file 4**. Mystery client information leaflet.

## Data Availability

The datasets supporting the conclusions of this article are available from the lead authors upon a reasonable request.

## Abbreviations

CARE: COVID-19 and Antimicrobial Resistance in East Africa
HATUA: Holistic Approach to Unravel Antibacterial Resistance in East Africa
GPS: Global Positioning System
Pharm: Pharmacy
DS: Drug shop
ADDO: Accredited drug dispensing outlet
AMR: Antimicrobial resistance
MC: Mystery client

## Footnotes

1 Suitably qualified persons are those with a pre-existing relevant qualification in nursing, pharmacy or clinical training—or other qualification approved by the Ministry of Health. To qualify for training, the potential dispenser must be one of the following: Pharmaceutical technician, Pharmaceutical assistant, Nursing officer, Nurse midwife, Clinical officer, Clinical assistant, Nursing assistant, or any other person seen as capable by the MoHSW after being advised by Tanzania Food and Drug Authority.
