# Supplementary material for: Antibiotic dispensing practices during COVID-19 and implications for antimicrobial resistance (AMR): parallel mystery client studies in Uganda and Tanzania
Source: Antimicrob Resist Infect Control. 2023 Feb 11;12:10. doi: 10.1186/s13756-022-01199-4 (PMC9919751; doi:10.1186/s13756-022-01199-4)
Supplement: Supplementary file 1 — Additional file 1. Copy of scripts given to MCs and supplementary analysis. [file 13756_2022_1199_MOESM1_ESM.docx]

**Supplementary Materials.**

1. **Mystery Client Scenario**

The mystery clients were provided with different scenarios and a number of prompts about how to respond to likely questions and suggestions from the drug sellers. A summary list of the key responses is presented below.

- Had not had these symptoms before, were not taking any drugs, did not have a prescription, had not been to a doctor and did not wish to have any test on the day.
- Had heard of COVID-19 before but was confused about what it is, its symptoms, and how to treat it.
- Requested amoxicillin (if the seller did not themselves suggest a pharmaceutical drug, because this was the drug they usually took for this kind of problem, and if amoxicillin was suggested by either client or seller), but that really, they preferred ‘something stronger because they were feeling a lot of pain.
- Wanted to buy just a couple of days of any drug offered, to ‘see if it works before buying more’.

Mystery clients asked once (for amoxicillin or something stronger/a few days’ worth) and did not attempt to ‘persuade’ the seller. They then took the seller’s advice, and bought whatever was recommended in whatever quantity the seller was prepared to dispense.

**Table S1:** Antibiotics sold to mystery clients presenting with COVID-19-like symptoms classified by WHO AWaRE grouping and country-specific relevance for treating secondary bacterial infection among patients with severe COVID-19 infection.

| **Antibiotics** | **Commonly recommended dosage** | **WHO AWaRe Classification** | **Relevance for treating severe COVID-19 infection^1^** |
| --- | --- | --- | --- |
| Amoxicillin | 500mg every 8 hrs for 5 to 7 days | Access | Yes |
| Ampicillin | 500mg every 6 hrs for 5 -7 days | Access | No |
| Ampiclox | 500mg every 8 hrs for 5 to 7 days | ^2^Access | No |
| Azithromycin | 500mg every 24hrs for 3 days | Watch | Yes |
| Cefalexin | 500mg every 12 hrs for 7-14 days | Access | No |
| Cephalexin | 500mg every 12 hrs for 7-10 days | Access | No |
| Ciprofloxacin | 500mg every 12 hrs for 7-10 days | Watch | No |
| Doxycycline | 500mg every 8 hrs for 5 to 7 days | Access | No |
| Erythromycin | 500mg every 8 hrs for 5-7 days | Watch | No |
| Metronidazole | 500mg every 8 hrs for 7-10 days | Access | No |
| Trimethoprim/ Sulfamethoxazole | 960mg every 12 hrs for 10-14 days | Access^2^ | No |
| Tetracycline | 500 mg every 6 hrs for 7- 10 days | Access | No |

^1 –^ Whether the antibiotic sold was approved within national treatment guideline as appropriate for treating secondary bacterial infections in patients with severe COVID-19; ^2^Trimethoprim/ Sulfamethoxazole is classified as Access by the WHO but Watch by the Tanzania Medicines & Medical Devices Authority (TMDA).

**Table S2.** Mean number of tablets sold during the MC encounters, by type of antibiotic, country and type of outlet

| **Drug Sold** | **Min. required tablets for the commonly recommended dosage** | **Drug Shop/ADDO** | | **Pharmacy** | |
| --- | --- | --- | --- | --- | --- |
|  |  | **Min-Max** | **Mean [Median]** | **Min-Max** | **Mean [Median]** |
| **Tanzania** | | | | | |
| Amoxicillin | 15 | 1-10 | 4.33 [5] | 2-10 | 5.6 [5] |
| Ampicillin | 20 | 2-10 | 4.46 [4] | 7-7 | 7 [7] |
| Ampiclox | 15 | 1-9 | 2.58 [2] | 2-10 | 4.33 [5] |
| Cefalexin | 14 | 2-2 | 2 [2] | 10-10 | 10 [10] |
| Ciprofloxacin | 14 | 5-5 | 5 [5] |  |  |
| Penicillin |  | 2-10 | 5.4 [5] | 5-5 | 5 [5] |
| Tetracycline | 28 | 2-4 | 3 [3] |  |  |
| Trimethoprim/ Sulfamethoxazole | 20 | 4-4 | 4 [4] |  |  |
|  |  |  |  |  |  |
| **Uganda** | | | | | |
|  |  |  |  |  |  |
| Amoxicillin | 15 | 3-30 | 13.5 [10] | 1-30 | 11.05 [10] |
| Ampicillin | 20 | 7-10 | 9.25 [10] | 5-12 | 8.71 [10] |
| Ampiclox | 15 | 10-20 | 15 [15] | 2-20 | 9.56 [10] |
| Azithromycin | 3 | 1-1 | 1 [1] | 1-3 | 2.5 [3] |
| Ciprofloxacin | 14 |  |  | 10-10 | 10 [10] |
| Erythromycin | 15 |  |  | 10-10 | 10 [10] |
| Metronidazole | 21 |  |  | 10-10 | 10 [10] |
| Penicillin |  |  |  | 5-20 | 10.03 [10] |
| Tetracycline | 28 |  |  | 10-10 | 10 [10] |
| Trimethoprim/ Sulfamethoxazole | 20 |  |  | 10-20 | 12.86 [10] |

Note: The number of tablets sold to the mystery clients was standardized using information on the dosage of the active ingredients (mg) for comparability across sites and sellers.
