# Supplementary material for: Antibiotic dispensing practices during COVID-19 and implications for antimicrobial resistance (AMR): parallel mystery client studies in Uganda and Tanzania
Source: Antimicrob Resist Infect Control. 2023 Feb 11;12:10. doi: 10.1186/s13756-022-01199-4 (PMC9919751; doi:10.1186/s13756-022-01199-4)
Supplement: Supplementary file 2 — Additional file 2. Copy of data collection instrument. [file 13756_2022_1199_MOESM2_ESM.pdf]

TZ CARE MCS FINAL

MCS

The Protocol Researcher - Please record the GPS 'on-site' before you approach the seller/enter the premises - do this as close as possible to the seller without drawing attention. Enter the premises/approach the seller and test the scenario - follow the scenario carefully and do not improvise. Once you have entered the GPS and obtained the drugs (or been refused) - retire somewhere private to fill in the rest of the data in EpiCollect before you visit the next drug seller. Do not visit pharmacies/drug shops close to one another to avoid suspicion. Aim at a minimum of 50 metres, or 10 blocks, from one shop to another. If the seller offers to call the Covid-19 taskforce on your behalf, tell them that you will take care of it yourself. Do not give them your telephone number or address.

Scenario part 1 1 Statements you make: You are supposed to be unwell with a temperature and a cough. Do not over-act but look unwell and cough a bit. 2 Symptoms you report: Tell the seller: I have a high temperature - it developed yesterday I have been coughing a lot - it is ok at the moment, but my throat is sore - the coughing was very bad this morning, last night and yesterday Food tastes strange when I eat - it is almost like it is tasteless - and I am not smelling things like I do normally Tell the seller you are seeking their advice - tell them you would normally buy amoxicillin for an illness like this - but this feels different and worse than usual - so what would they recommend?

Scenario part 2 3 Answers to questions you may be asked: If you are asked questions about your illness (and you may not be) - answer using the appropriate response below - you may need to adapt them a little - but keep the key facts the same: NoI have not had these symptoms before - it has come on in the last day or two NoI am not taking something for these symptoms NoI am not taking any other drugs NoI do not have a prescription NoI have not been to see a doctor for this NoI am not pregnant [if researcher is female] NoI do not wish to have any kind of test today to see what the problem might be - I do not have the time/money/I don't feel able (whatever works that allows the client not to be tested

Scenario part 3 4 Answers to give if you are asked directly about your knowledge of Corona virus/Covid-19: Yes - I have heard of it - but I am confused (confused about what it is, about what the symptoms are, about how to treat it, about what to do if you think you have it) 5 Response to being offered a drug: If you are offered only advice - or If you are offered something that does not sound like an antibiotic ask - Can't I have some Amoxicillin for this? I usually take Amoxicillin for this kind of problem - or even if you have something stronger because I am feeling a lot of pain? If you are offered an antibiotic - accept it

Scenario part 4 6 Attempt to by a half dose/course: For any drug you agree to buy - attempt to buy just a couple of days' worth - say you want to see if it works before you buy more 7 Take the seller's advice: After the conversation (see above) you should eventually take the sellers advice, i.e. one or more of the following: Accept you do not need antibiotics if that is what is advised Buy whatever is eventually recommended Buy a half course of the recommended drug if the seller will allow that Buy a full course of the drugs recommended if the seller says they will not sell a half course Accept you should go for a test if that is advised, but say you will arrange it yourself

Location of antibiotic seller (record just BEFORE you enter premises)

Enter street address or brief location description

Metadata about the seller/premises

Date of Study

Time of study

YOUR Name (researcher)

Type of seller

- ○ Hospital pharmacy
- ○ Pharmacy
- ○ ADDO / Duka la Dowa / or similar
- ○ Alternative medicine (e.g. Chinese medicine)
- ○ Traditional medicine
- ○ Agro vet Pharmacy
- ○ Other

If Other ticked above, please describe type of seller

Type of ownership

- ○ Government
- ○ Private

Type of affiliation

- ○ Hospital/clinic-associated
- ○ Not associated with a hospital/clinic (i.e., a freelance shop)

Size of the pharmacy

- ○ Large/wholesale pharmacy
- ○ Small drug shop

Rate the seller's premises - in terms of the likelihood that dirt, moisture, temperature or pests might compromise the safe storage of drugs

- ○ POOR - among the worst (e.g. not clean, not well organized/ordered, and/or unlikely to keep dust, moisture or pests out)
- ○ ADEQUATE - about average (e.g. sufficiently clean, well-ordered and organized, structurally sound premises)
- ○ EXCELLENT - among the best (e.g. extremely clean, highly professionally organized, the premises is an excellent environment to store drugs safely)

Did the premises offer either or both of these additional services?

- ☐ Presence of diagnostic services in the premises
- ☐ Availability of other non medical items in the outlet

Did the drug seller wear an ID badge?

- ○ Yes
- ○ No

If there were other employees - did they have ID badges?

- ○ Yes
- ○ No

Were licenses/certificates of qualifification displayed?

- ○ Yes
- ○ No

Questions you were asked by the seller

1. Did the seller ask if you had experienced these symptoms before?

- ○ No
- ○ Yes

2. Did the seller ask you if you were pregnant?

- ○ Not applicable (mystery client male – or older woman)
- ○ No
- ○ Yes

3. Did the seller ask if you were taking any other medication?

- ○ No
- ○ Yes

4. Did the seller ask if you had seen a doctor?

- ○ No
- ○ Yes

5. Did the seller ask if you had a prescription?

- ○ No
- ○ Yes

Advice you were given

6. Did the seller mention Covid-19 - suggest they think you might have Covid-19?

- ○ No
- ○ Yes

7. Did the seller advise you to treat yourself at home with remedies that you already have, such as herbs, steam baths, etc.?

- ○ No
- ○ Yes

8. Did the seller recommend that you eat immunity-boosting foods - or give similar nutrition-related advice?

- ○ No
- ○ Yes

9. Did the seller suggest that you call the toll-free Covid-19 taskforce helpline?

- ○ N/A
- ○ No
- ○ Yes

10. Did the seller offer to call the toll-free Covid-19 taskforce helpline on your behalf?

- ○ N/A
- ○ No
- ○ Yes

11. Did the seller recommend that you go to a doctor/hospital for a check-up/to receive medical attention?

- ○ No
- ○ Yes

12. Did the seller advise you to get tested for Covid-19?

- ○ No
- ○ Yes

13. Did the seller advise you to self-isolate until your symptoms clear?

- ○ No
- ○ Yes

14. Did the seller advise you to maintain social distance?

- ○ No
- ○ Yes

15. Did the seller advise you to wear a face mask?

- ○ No
- ○ Yes

16. Did the seller suggest that you do not need to do anything - just wait for the symptoms to clear?

- ○ No
- ○ Yes

17. Did the seller recommend that you buy a medication/drug/treatment?

- ○ No
- ○ Yes

Did the seller give you any other advice? If yes, please describe advice given.

19. Did the seller sell you a herbal/traditional/alternative treatment?

- ○ No
- ○ Yes

What is the name of the herbal/traditional/alternative drug you were sold?

Take a photo of the front of the drug packaging (or drug itself if no packaging)

Take a photo of the back of the drug packaging (or drug itself if no packaging)

21. Did the seller sell you a pharmaceutical drug?

- ○ No
- ○ Yes (you will skip to Q24)

22. If you were NOT sold a pharmaceutical drug, did the seller explain why it was not appropriate for your symptoms?

- ○ No
- ○ Yes

23. If you were NOT sold a pharmaceutical drug, record any advice you were given by the seller in relation to treating Covid-19 with pharmaceutical drugs/antibiotics. Free text or write NONE:

24. If you were sold a pharmaceutical drug - what is the name of the drug. If more than one, enter the name of the first drug you were sold.

· Drug 1: take a photo of the front of the drug packaging

· Drug 1: take a photo of the back of the drug packaging

Were you sold more than one type of drug?

- ○ No
- ○ Yes

If you were sold a second type of drug, enter the name of the drug here

If 2 drugs sold, take a photo of the front of the drug packaging

If 2 drugs sold, take a photo of the back of the drug packaging

25. If you were sold a pharmaceutical drug - did the seller recommend pharmaceutical drugs with no suggestion from you?

- ○ No
- ○ Yes

26. If you had to ask for a pharmaceutical drug, which of the following was true?

- ○ Not applicable - – I failed to ask for a pharmaceutircal drug
- ○ The seller agreed to sell it without further comments
- ○ The seller agreed to sell it, but advised you that it is probably not suitable for the type of symptoms you described
- ○ The seller refused to sell it because it is not suitable for the symptoms you described

27. If you were sold a pharmaceutical drug, which of the following was true?

- ○ The seller sold a half course with no further questions

- ○ The seller advised to buy a full course, but sold a half course anyway
- ○ The seller insisted that you buy a full course

28. Did the seller give clear instructions (oral or written) that you should buy a full course (even if they eventually sold a half course)?

- ○ No
- ○ Yes

29. Did the seller give clear instructions (oral or written) that you should normally finish a full course (even if they eventually sold a half course)?

- ○ No
- ○ Yes

30. If instructions were given either in written or oral format, please write what was advised. If written instructions provided, please take a photo as well

[ ]

30. If written instructions provided, please take a photo

[ ]

31. If you were sold a pharmaceutical drug, how many tablets were you sold? If you were sold more than one type of drug, enter the number of tablets only for first type of drug here.

[ ]

If you were sold two different types of pharmaceutical drugs, how many tablets were you sold of the second type?

[ ]

32. If you were sold a pharmaceutical drug - what regimen was advised for the first type of drug sold? (Either written on the drug packaging, or specified by the drug seller: e.g., one pill twice a day for a week)

[ ]

If you were sold two different types of pharmaceutical drugs, what regimen was advised? (Either written on the drug packaging, or specified by the drug seller: e.g., one pill twice a day for a week)

[ ]

Covid-19 prevention measures The mystery client should observe and note the following:

---

33. Was social distancing observed by the seller (i.e. did they avoid close proximity and not touch you e.g. by remaining behind a counter and not shaking hands)?

- ○ No
- ○ Yes

34. If social distancing was observed by the seller, which of the following was true. Choose one:

- ○ Social distancing seemed intentional / a conscious policy (e.g. seller said something / there were clear signs / seller kept their distance
- ○ · Social distancing occurred but seemed more accidental or circumstantial than a conscious policy (e.g. the seller was behind a counter)

35. Did you observe social distancing of customers?

- ○ Not applicable [no other customers in the shop]
- ○ Mixed [some customers observed social distancing and others didn’t]
- ○ No
- ○ Yes

36. If you observed social distancing of customers, which of the following was true. Choose one:

- ○ Social distancing seemed intentional (e.g., the customers seemed to deliberately maintain distance between one another)
- ○ Social distancing occurred but seemed more accidental or circumstantial than a conscious decision (e.g., there were very few customers in the shop)

37. Was the seller wearing a mask?

- ○ No
- ○ Yes

COVID-19 prevention measures

38. Was any employee wearing a mask?

- ○ Not applicable (no other employees in the shop)
- ○ Mixed [some employees wore masks and others didn't)
- ○ No
- ○ Yes

39. Was any customer wearing a mask?

- ○ Not applicable (no other customers in the shop)
- ○ Mixed [some customers wore masks and others didn't)
- ○ No
- ○ Yes

40. Was there a sanitizer outside or inside the drug shop/pharmacy for client use?

- ○ No
- ○ Yes

41. Was there a hand-washing facility outside or inside the drug shop/pharmacy for client use?

- ○ No
- ○ Yes

42. Did the drug seller seem shocked or alarmed by the mention of Covid-19-like symptoms?

- ○ No
- ○ Yes

43. Was there any health education information related to Covid-19 inside or outside the drug shop/pharmacy?

- ○ No
- ○ Yes

44. If there was health education information related to Covid-19 inside or outside, which of the following was true? (Select all that apply)

- ☐ It looked official (government, WHO or similar) and was in English
- ☐ It looked official (government, WHO or similar) and was in a local language
- ☐ It looked unofficial (homemade) and was in English
- ☐ It looked unofficial (homemade) and was in a local language
- ☐ It was sponsored by a private company/drug manufacturer

45. Did you observe any health education information related to Antibiotics use inside or outside the drug shop/pharmacy?

- ○ No
- ○ Yes

46. If there was health education information related to Antibiotics use inside or outside, which of the following was true? (Select all that apply)

- ☐ It looked official (government, WHO or similar) and was in English
- ☐ It looked official (government, WHO or similar) and was in a local language
- ☐ It looked unofficial (homemade) and was in English
- ☐ It looked unofficial (homemade) and was in a local language
- ☐ It was sponsored by a private company/drug manufacturer

47. In free text - Please make any further notes that you feel are relevant to explain: (a) your interaction; (b) the seller; (c) the premises/stall; (d) the drugs/products available; (e) observations about other interactions witnessed; (f) Anything else

SAVE entry in EpiCollect and upload
