# Supplementary material for: Antibiotic dispensing practices during COVID-19 and implications for antimicrobial resistance (AMR): parallel mystery client studies in Uganda and Tanzania
Source: Antimicrob Resist Infect Control. 2023 Feb 11;12:10. doi: 10.1186/s13756-022-01199-4 (PMC9919751; doi:10.1186/s13756-022-01199-4)
Supplement: Supplementary file 3 — Additional file 3. Checklist for reporting research using simulated patient. [file 13756_2022_1199_MOESM3_ESM.pdf]

Checklist for Reporting research using Simulated Patient methodology (CRiSP)

Section	Item	Description	Page Number
Title and Background	1	Include the term simulated patient or some variant (e.g. mystery shopper).	5
	2	Describe the rationale, theory or goal behind using simulated patient methodology.	5
	3	Report the study design used (e.g. cross-sectional, case-control, randomised controlled trial, etc).*	5
Simulated Patients	4a	Report the number of simulated patients used in the study.	5
	4b	If more than one simulated patient was used, describe methods used to minimise variability between simulated patients.	6
	5	Report demographics of the simulated patient(s) (include age, gender, qualifications (e.g. student, academic) and behaviour characteristics relevant to the scenario).	5
	6	Describe what was done during training sessions for simulated patients (e.g. role-play scenarios, etc.).	6
	7	Actual: If simulated patient adherence or fidelity was assessed, describe the extent to which the scenario was delivered as planned.	6
Simulated Patient Scenarios	8a	Describe how the scenarios were developed (including who they were developed by).	6
	8b	Describe if any guidelines (i.e. best practice guidelines) were used in development of the scenarios or if the scenarios were validated.	6
	9a	Give details about the scenario(s) used. Include any patient characteristics, patient prompts, scripts, props (e.g. prescriptions, medical devices), etc.	S1
	9b	Describe any flexibility in scenarios or scripts to allow simulated patients to adapt based on participant responses.	6
	10a	Materials: Describe any physical or informational materials used in the intervention, including those provided to participants or used in intervention delivery or in training of intervention providers. Provide information on where the materials can be accessed (e.g. online appendix, URL).	S4
	10b	Include a copy of any scripts or material given to simulated patients.	S1
	11	Describe any intervention completed prior to the simulated patient assessment. Include procedures, activities, and/or processes (e.g. training sessions for health professionals).	N/A

* CRiSP is designed to be an extension; additional checklists should be consulted depending on the study type.

Checklist for Reporting research using Simulated Patient methodology (CRiSP)

Simulated Patient Scenarios	12	Procedures: Describe each of the procedures, activities, and/or processes used in the intervention, including any enabling or support activities.	N/A
	13	If the simulated patient assessment was modified (e.g., changes in personnel, assessment rubric, patient history or problems, etc.), describe these changes (what, why, when, and how).	N/A
	14	Describe any procedures that followed the simulated patient assessment (e.g. debriefs, performance feedback), including how (face to face, phone, etc.) and when these were conducted.	N/A
	15	Describe any procedures if simulated patients were identified by participants.	N/A
Data Collection	16	Report how many simulated patient visits were conducted (include the planned number of visits, the number of actual completed visits, the number of visits per SP, number per scenario, number per health services provider e.g. pharmacy).	7
	17	Describe the mode(s) of delivery of the simulated patient assessment (e.g., face-to-face, telephone, internet, text, live, asynchronous, etc.).	5
	18	Describe the data collection procedure (e.g. data collection form, audio recording, telephone calls etc.).	5
	19	Describe how any data collection forms were created and validated (include a copy of any data collection forms if possible).	S2
	20	Describe when the data was collected by the simulated patient (i.e. during the visit, immediately after, etc.).	6
	21	Address ways to avoid or minimise recall bias (e.g. the time taken to record data, use of audiotaping, use of an observer, etc).	6
	22	Report any conflicts of interest for assessors (e.g. if a simulated patient is a student assessing a colleague or preceptor)	26
Ethics	23a	Describe any ethics approval processes, consent from participants and ways of maintaining confidentiality.	7, 25
	23b	Explain how participants were informed about being assessed using covert methods. If they were not, justify this.	7

* CRiSP is designed to be an extension; additional checklists should be consulted depending on the study type.
