# Supplementary material for: Antibiotic dispensing practices during COVID-19 and implications for antimicrobial resistance (AMR): parallel mystery client studies in Uganda and Tanzania
Source: Antimicrob Resist Infect Control. 2023 Feb 11;12:10. doi: 10.1186/s13756-022-01199-4 (PMC9919751; doi:10.1186/s13756-022-01199-4)
Supplement: Supplementary file 4 — Additional file 4. Mystery client information leaflet. [file 13756_2022_1199_MOESM4_ESM.pdf]

Mystery Client Information Leaflet (Example)

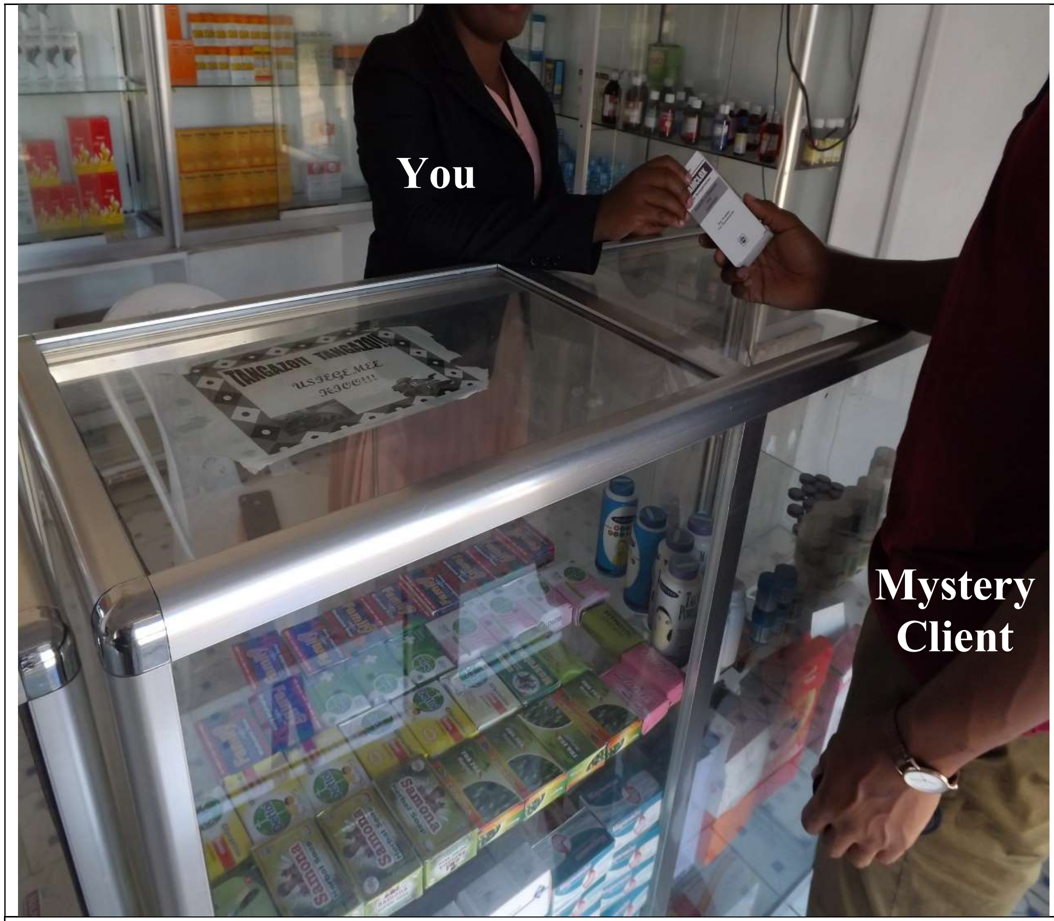

Mystery Client Survey

In your area sometime in the next 90 days

INSERT COUNTRY SPECIFIC INSTITUTIONAL NAME / LOGO – HERE

Is conducting research on Drug resistance.

Research Aims:
To investigate the how the treatment seeking behaviour of patients and the provision practices of antibiotic sellers affects the development of antimicrobial resistance in patients with UTI.

What do you need to do?
Inform all your staff of the survey – then carry on as normal.

What will happen?
A researcher will come unannounced in the next 90 days to use your services and record what happened.

Do you need to worry?
No. Findings will be used for research only. No personal data will be collected and neither your store or behaviour will be directly identified to the authorities or in any publication
